# Kale Extract Increases Glutathione Levels in V79 Cells, but Does not Protect Them Against Acute Toxicity Induced by Hydrogen Peroxide

**DOI:** 10.3390/molecules17055269

**Published:** 2012-05-07

**Authors:** Fátima Fernandes, Carla Sousa, Federico Ferreres, Patrícia Valentão, Fernando Remião, José A. Pereira, Paula B. Andrade

**Affiliations:** 1 REQUIMTE/Laboratório de Farmacognosia, Departamento de Química, Faculdade de Farmácia, Universidade do Porto, Rua de Jorge Viterbo Ferreira, nº 228, 4050-313 Porto, Portugal; Email: mfgfernandes@gmail.com (F.F.); csousa@ff.up.pt (C.S.); valentao@ff.up.pt (P.V.); 2 Research Group on Quality, Safety and Bioactivity of Plant Foods, Department of Food Science and Technology, CEBAS (CSIC), P.O. Box 164, Campus University Espinardo, Murcia 30100, Spain; Email: federico@cebas.csic.es; 3 REQUIMTE/Laboratório de Toxicologia, Departamento de Ciências Biológicas, Faculdade de Farmácia, Universidade do Porto, Rua de Jorge Viterbo Ferreira, nº 228, 4050-313 Porto, Portugal; Email: remiao@ff.up.pt; 4 CIMO/Escola de Agricultura, Instituto Politécnico de Bragança, Campus Sta Apolónia, Apt. 1171, 5301-854 Bragança, Portugal; Email: jpereira@ipb.pt

**Keywords:** *Brassica oleracea* var.* acephala*, *Pieris brassicae*, phenolic compounds, V79 cells, oxidative stress, glutathione status

## Abstract

This study aims to evaluate the antioxidant potential of extracts of *Brassica oleracea* L. var. *acephala* DC. (kale) and several materials of *Pieris brassicae* L., a common pest of *Brassica* cultures using a cellular model with hamster lung fibroblast (V79 cells) under quiescent conditions and subjected to H_2_O_2_-induced oxidative stress. Cytotoxicity was evaluated by 3-(4,5-dimethylthiazol-2-yl)-2,5-diphenyl tetrazolium bromide (MTT) assay and glutathione was determined by the 5,5'-dithiobis(2-nitrobenzoic acid) (DTNB)-oxidized glutathione (GSSG) reductase recycling assay. The phenolic composition of the extracts was also established by HPLC-DAD. They presented acylated and non acylated flavonoid glycosides, some of them sulfated, and hydroxycinnamic acyl gentiobiosides. All extracts were cytotoxic by themselves at high concentrations and failed to protect V79 cells against H_2_O_2_ acute toxicity. No relationship between phenolic composition and cytotoxicity of the extracts was found. Rather, a significant increase in glutathione was observed in cells exposed to kale extract, which contained the highest amount and variety of flavonoids. It can be concluded that although flavonoids-rich extracts have the ability to increase cellular antioxidant defenses, the use of extracts of kale and *P. brassicae* materials by pharmaceutical or food industries, may constitute an insult to health, especially to debilitated individuals, if high doses are consumed.

## 1. Introduction

Several epidemiological studies have indicated that regular consumption of *Brassica* vegetables is strongly associated with a reduced risk of developing certain cancers [1]. In general, the consumption of fruits and vegetables can bring beneficial health effects due to the presence of a wide array of phytochemicals. Phenolic compounds are claimed to possess antioxidant properties and therefore can help in the prevention of chronic diseases, such as atherosclerosis, diabetes, cardiovascular diseases and ischemia and of neurodegenerative disorders like Alzheimer’s and Parkinson’s [2,3].

In fact, studies using cell cultures demonstrated that flavonoids like kaempferol, quercetin and their glycosides have antioxidant activity. In contrast to their antioxidant activity, phenolics also have the potential to act as pro-oxidants under certain conditions. The pro-oxidant properties of flavonoids and other polyphenols could contribute to tumor cell apoptosis and cancer chemoprevention induced by oxidant species [4].

*Brassica oleracea* L. varieties, namely kale (*Brassica oleracea* L. var. *acephala* DC.), have been intensively studied and are an important dietary source of bioactive compounds, including glucosinolates and phenolics, like flavonols and hydroxycinnamic acid derivatives [5,6,7]. 

The study of the role of several classes of compounds in shaping insect-plant relationships has known a great impulse over the last few years. Our group described the role of phenolic compounds in the modulation of the feeding behavior of herbivore organisms, such as *Pieris brassicae* L. (Lepidoptera: Pieridae), a common pest of *Brassica* cultures. This insect revealed ability to sequester, metabolize and excrete phenolics obtained from distinct host plants [6,8,9,10,11,12]. Furthermore, the extracts of *P. brassicae* materials (butterfly, larvae and its excrements) reared on kale proved to have a better antioxidant potential than host plant, depending on the radical species, in cell free systems [6]. As so, *P. brassicae* may have interest as a source of antioxidants. 

**Figure 1:**
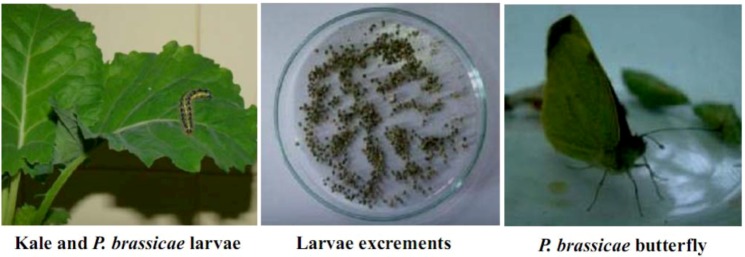
Kale and *P. brassicae* materials used in this study.

It is known that the antioxidant activity of a given matrix is dependent on the test system used [13]. Extracts that reveal high antioxidant potential in cell free systems can be either protective or toxic in cellular assays, depending on the extract concentration and cellular conditions [14,15]. So, this work intended to characterize the antioxidant capacity of kale and materials of *P. brassicae* (Figure 1) reared on this plant in hamster lung fibroblast (V79 cells) subjected to hydrogen peroxide (H_2_O_2_)-induced oxidative stress. To assess the effects in V79 cells under quiescent conditions and exposed to H_2_O_2_, cellular viability and glutathione (GSH) status were evaluated by the 3-(4,5-dimethylthiazol-2-yl)-2,5-diphenyl tetrazolium bromide (MTT) reduction assay and 5,5'-dithiobis(2-nitrobenzoic acid) (DTNB)-oxidized glutathione (GSSG) reductase recycling assay, respectively. Additionally, phenolic composition of the extracts was determined by high performance liquid chromatography with diode-array detection (HPLC-DAD) in order to establish possible relations with the cellular effects.

## 2. Results

### 2.1. Chemical Composition

#### 2.1.1. HPLC-DAD Phenolic Compounds Qualitative Analysis

**Figure 2:**
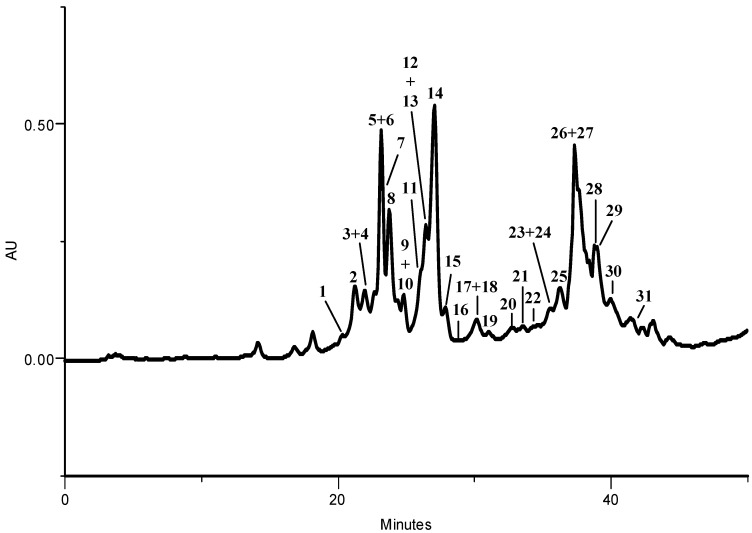
HPLC-DAD phenolic profile of the methanolic extract of *B. oleracea* var. *acephala* leaves. Detection at 330 nm. Peaks: (**1**) quercetin-3-*O*-sophorotrioside-7-*O*-glucoside, (**2**) quercetin-3-*O*-sophoroside-7-*O*-glucoside, (**3**) kaempferol-3-*O*-(methoxy-caffeoyl)sophoroside-7-*O*-glucoside, (**4**) quercetin-3-*O*-sophoroside-7-*O*-diglucoside, (**5**) kaempferol-3-*O*-sophoroside-7-*O*-glucoside, (**6**) kaempferol-3-*O*-(caffeoyl)sophoroside-7-*O*-glucoside, (**7**) quercetin-3-*O*-(sinapoyl)sophoroside-7-*O*-glucoside, (**8**) kaempferol-3-*O*-sophoroside-7-*O*-diglucoside, (**9**) isorhamnetin-3-*O*-sophoroside-7-*O*-glucoside, (**10**) quercetin-3-*O*-(feruloyl)sophoroside-7-*O*-glucoside, (**11**) quercetin-3-*O*-(feruloyl)-sophoroside-7-*O*-diglucoside, (**12**) kaempferol-3-*O*-(sinapoyl)sophoroside-7-*O*-glucoside, (**13**) kaempferol-3-*O*-(sinapoyl)sophoroside-7-*O*-diglucoside, (**14**) kaempferol-3-*O*-(feruloyl)sophoroside-7-*O*-glucoside, (**15**) kaempferol-3-*O*-(feruloyl)sophoroside-7-*O*-diglucoside, (**16**) kaempferol-3-*O*-(*p*-coumaroyl)sophoroside-7-*O*-glucoside, (**17**) kaempferol-3-*O*-(*p*-coumaroyl)sophoroside-7-*O*-diglucoside, (**18**) kaempferol-3-*O*-gentio-bioside-7-*O*-glucoside, (**19**) kaempferol-3-*O*-gentiobioside-7-*O*-diglucoside, (**20**) isorhamnetin-3-*O*-gentiobioside-7-*O*-glucoside, (**21**) quercetin-3-*O*-(sinapoyl)sophoroside, (**22**) quercetin-3-*O*-(feruloyl)sophoroside, (**23**) quercetin-3-*O*-sophoroside, (**24**) kaempferol-3-*O*-(*p*-coumaroyl)gentiobioside-7-*O*-glucoside, (**25**) kaempferol-3-*O*-(sinapoyl)sophoroside, (**26**) kaempferol-3-*O*-sophoroside, (**27**) kaempferol-3-*O*-(feruloyl)-sophoroside, (**28**) disinapoyl-gentiobioside, (**29**) sinapoyl,feruloyl-gentiobioside, (**30**) diferuloyl-gentiobioside, (**31**) disinapoyl,feruloyl-gentiobioside.

**Figure 3:**
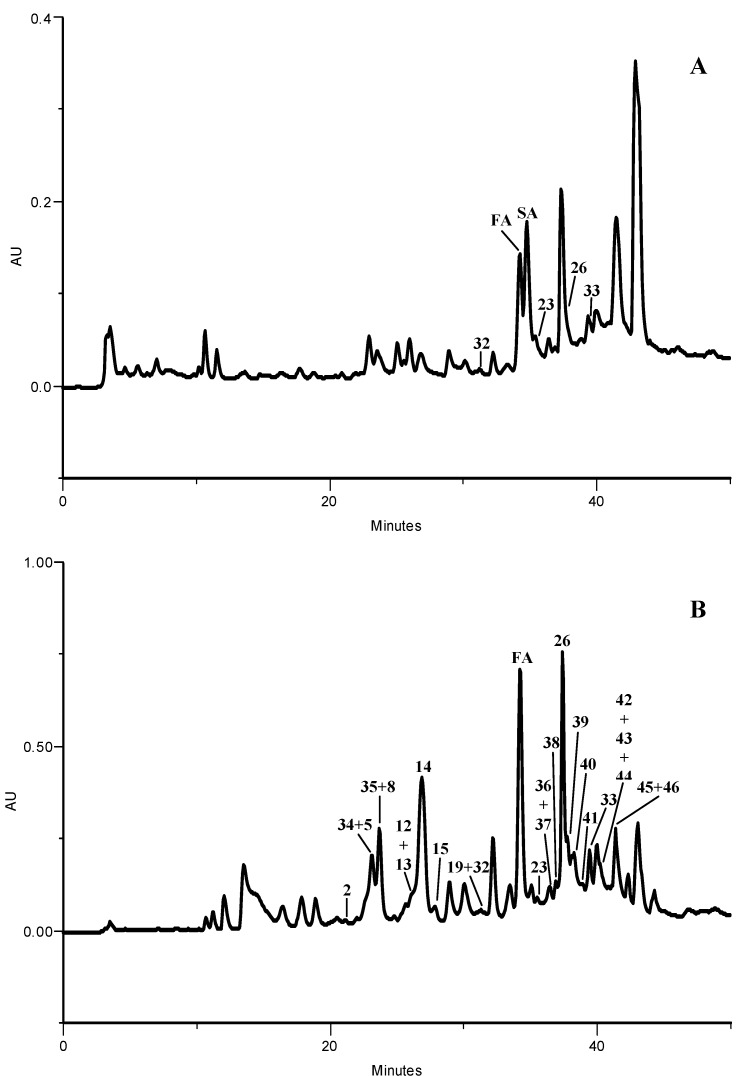
HPLC-DAD phenolic profiles of *P. brassicae* larvae (**A**) and excrement (**B**) methanolic extracts. Detection at 330 nm. Peaks: **2**, **5**, **8**, **12**–**15**, **19**, **23** and **26** see Figure 2. (**FA**) Ferulic acid, (**SA**) sinapic acid, (**32**) kaempferol-3-*O*-sophoroside sulfate, (**33**) kaempferol-3-*O*-glucoside sulfate, (**34**) kaempferol-3-*O*-sophorotrioside-7-*O*-glucoside, (**35**) kaempferol-3-*O*-sophorotrioside-7-*O*-diglucoside; (**36**) kaempferol-3-*O*-(sinapoyl)sophorotrioside, (**37**) kaempferol-3-*O*-sophorotrioside, (**38**) kaempferol-3-*O*-(feruloyl)sophorotrioside, (**39**) isorhamnetin-3-*O*-sophoroside, (**40**) kaempferol-3-*O*-(*p*-coumaroyl)sophorotrioside, (**41**) quercetin-3-*O*-glucoside sulfate, (**42**) kaempferol-3-*O*-gentiobioside, (**43**) kaempferol-3-*O*-glucoside, (**44**) isorhamnetin-3-*O*-gentiobioside, (**45**) kaempferol-3-*O*-(feruloyl)sophoroside, (**46**) kaempferol-3-*O*-(*p*-coumaroyl)sophoroside.

In order to characterize the methanolic extracts of kale and of *P. brassicae* butterflies, larvae and its excrements, phenolic compounds were identified by HPLC-DAD. The extracts from kale (Figure 2), *P. brassicae* larvae (Figure 3A) and their excrements (Figure 3B) contained four groups of phenolic compounds: non acylated flavonoid glycosides (compounds **1**, **2**, **4**, **5**, **8**, **9**, **18**–**20**, **23**, **26**, **32**–**35**, **37**, **39**, **41**–**44**), flavonoid glycosides acylated with hydroxycinnamic acids (**3**, **6**, **7**, **10**–**17**, **21**, **22**, **24**, **25**, **27**, **36**, **38**, **40**, **45** and **46**), hydroxycinnamic acyl gentiobiosides (**28**–**31**) and free hydroxycinnamic acids (**FA** and **SA**). 

In the methanolic extract of kale leaves sixteen kaempferol derivatives, nine quercetin derivatives, two isorhamnetin glycosides and four phenolic acids heterosides were found (Figure 2). The phenolic profile of *P. brassicae* larvae was composed by six compounds, including free ferulic and sinapic acids (**FA** and **SA**, respectively), two non acylated flavonoid glycosides (**23**, **26**) and two sulfate derivatives of non acylated flavonoid glycosides (**32**, **33**) (Figure 3A). Concerning *P. brassicae* excrements, six non-acylated glycosyl flavonols (**2**, **5**, **8**, **19**, **23** and **26)** were detected (Figure 3B). Other non acylated glycosides present in excrements, and not detected in kale leaves, were kaempferol (**32**–**35**, **37**, **42** and **43**), quercetin (**41**) and isorhamnetin (**39** and **44**) derivatives (Table 1). In addition, as it happened with the larvae methanolic extract, flavonol sulfated derivatives were also noted (**32**, **33** and **41**) (Figure 3B, Table 1). No phenolic compound was characterized in *P. brassicae* butterfly methanolic extract.

**Table 1:** Quantification of phenolic compounds in methanolic extracts of *B. oleracea* var. *acephala* and *P. brassicae* materials (mg/kg, dry basis) ^a^.

	Compound	Kale	Larvae	Excrements
**1**	Quercetin-3-*O*-sophtr-7-*O*-gluc	12.0 (0.1)	-	-
**2**	Quercetin-3-*O*-soph-7-*O*-gluc	24.3 (1.0)	-	nq
**3**	Kaempferol-3-*O*-(methoxycaffeoyl)soph-7-*O*-gluc +	244.1 (15.5)	-	-
**4**	Quercetin-3-*O*-soph-7-*O*-digluc		-	-
**34**	Kaempferol-3-*O*-sophtr-7-*O*-gluc +	-	-	1.4 (0.0)
**5**	Kaempferol-3-*O*-soph-7-*O*-gluc +	310.9 (0.1)	-	
**6**	Kaempferol-3-*O*-(caffeoyl)soph-7-*O*-gluc +		-	-
**7**	Quercetin-3-*O*-(sinapoyl)soph-7-*O*-gluc		-	-
**35**	Kaempferol-3-*O*-sophtr-7-*O*-digluc +	-	-	5.4 (0.1)
**8**	Kaempferol-3-*O*-soph-7-*O*-digluc	75.1 (8.2)	-	
**9**	Isorhamnetin-3-*O*-soph-7-*O*-gluc +	34.8 (2.6)	-	-
**10**	Quercetin-3-*O*-(feruloyl)soph-7-*O*-gluc		-	-
**11**	Quercetin-3-*O*-(feruloyl)soph-7-*O*-soph +	883.0 (97.8)	-	-
**12**	Kaempferol-3-*O*-(sinapoyl)soph-7-*O*-gluc +		-	7.9 (0.1)
**13**	Kaempferol-3-*O*-(sinapoyl)soph-7-*O*-soph		-	
**14**	Kaempferol-3-*O*-(feruloyl)soph-7-*O*-gluc	581.6 (0.7)	-	10.5 (0.1)
**15**	Kaempferol-3-*O*-(feruloyl)soph-7-*O*-soph	70.4 (8.8)	-	1.1 (0.1)
**16**	Kaempferol-3-*O*-(*p*-coumaroyl)soph-7-*O*-gluc	nq	-	-
**17**	Kaempferol-3-*O*-(*p*-coumaroyl)soph-7-*O*-soph +	159.5 (12.3)	-	-
**18**	Kaempferol-3-*O*-gent-7-*O*-gluc		-	-
**19**	Kaempferol-3-*O*-gent-7-*O*-digluc +	29.7 (4.7)	-	3.8 (0.7)
**32**	Kaempferol-3-*O*-digluc sulfate	-	nq	
**20**	Isorhamnetin-3-*O*-gent-7-*O*-gluc	101.6 (9.6)	-	-
**21**	Quercetin-3-*O*-(sinapoyl)soph	16.8 (0.1)	-	-
**22**	Quercetin-3-*O*-(feruloyl)soph	16.3 (3.6)	-	-
**FA**	Ferulic acid	-	8.3 (0.9)	3.0 (0.0)
**23**	Quercetin-3-*O*-soph +	31.4 (0.9)	16.4 (0.8)	1.1 (0.0)
**24**	Kaempferol-3-*O*-(*p*-coumaroyl)gent-7-*O*-gluc		-	-
**SA**	Sinapic acid	-	191.0 (18.1)	-
**25**	Kaempferol-3-*O*-(sinapoyl)soph	133.5 (0.9)	-	-
**26**	Kaempferol-3-*O*-soph +	960.8 (35.4)	55.8 (1.9)	2.6 (0.2)
**27**	Kaempferol-3-*O*-(feruloyl)soph		-	-
**28**	Disinapoyl-gent +	92.2 (6.3)	-	-
**29**	Sinapoyl,feruloyl-gent		-	-
**30**	Diferuloyl-gent	1.6 (0.1)	-	-
**31**	Disinapoyl,feruloyl-gent	nq	-	-
**33**	Kaempferol-3-*O*-gluc sulfate	-	nq	3.0 (0.0)
**36**	Kaempferol-3-*O*-(sinapoyl)sophtr +	-	-	1.2 (0.3)
**37**	Kaempferol-3-*O*-sophtr	-	-	
**38**	Kaempferol-3-*O*-(feruloyl)sophtr	-	-	2.4 (0.1)
**39**	Isorhamnetin-3-*O*-soph	-	-	0.3 (0.0)
**40**	Kaempferol-3-*O*-(*p*-coumaroyl)sophtr	-	-	0.1 (0.0)
**41**	Quercetin-3-*O*-gluc sulfate	-	-	nq
**42**	Kaempferol-3-*O*-gent	-	-	nq
**43**	Kaempferol-3-*O*-gluc	-	-	nq
**44**	Isorhamnetin-3-*O*-gent	-	-	nq
**45**	Kaempferol-3-*O*-(feruloyl)soph +	-	-	5.5 (0.3)
**46**	Kaempferol-3-*O*-(*p*-coumaroyl)soph	-	-	
	Σ	**3779.6**	**271.5**	**49.3**

^a^ Results are expressed as mean (standard deviation) of three determinations; Σ, sum of the determined phenolic compounds; nq: not quantified; -: not detected. No result means the compound was quantified with the previous one; sophtr: sophorotriose; soph: sophorose; gluc: glucose; digluc: diglucose; gent: gentiobiose.

#### 2.1.2. Phenolic Compounds Quantitative Analysis

**Figure 4:**
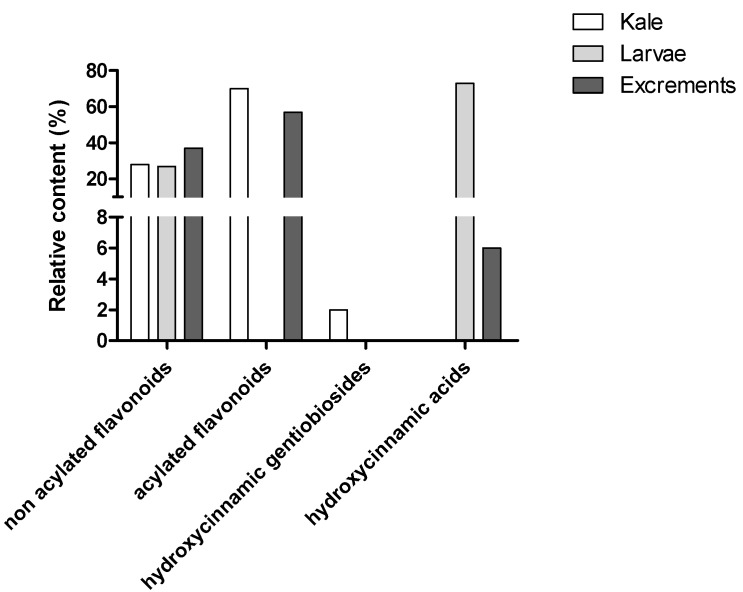
Relative content of the different classes of phenolic compounds in methanolic extracts of *B. oleracea* var. *acephala* and *P. brassicae* materials.

To better characterize the extracts used in cellular assays, the phenolic compounds were also quantified by HPLC-DAD (Table 1). The highest phenolics content was found in kale leaves extract (ca. 3780 mg/Kg phenolic compounds), followed by *P. brassicae* larvae and their excrements, with 272 and 49 mg/Kg, respectively (Table 1). The pair **26** plus **27**, the group **11**, **12** plus **13** and compound **14** were the main phenolics in kale, representing ca. 25%, 23% and 15% of total compounds, respectively (Table 1). Concerning *P. brassicae* larvae, the main compound was sinapic acid, corresponding to 70% of total identified compounds. Kaempferol-3-*O*-sophoroside (compound **26)** was the flavonol present at higher levels, representing ca. 21% of total phenolics (Table 1). In *P. brassicae* excrement extract, compound **14** and the pair **12** plus **13** were the major ones (ca. 21% and 16%, respectively) (Table 1). Concerning the different classes of flavonoids, it can be seen that the three analyzed matrices contain similar relative amounts of non acylated flavonoid glycosides, but differ in flavonoid derivatives acylated with hydroxycinnamic acids, which are absent in larvae extracts (Figure 4). With respect to phenolic acids, kale only contains hydroxycinnamic acyl gentiobiosides, while *P. brassicae* materials (larvae and excrements) just present free hydroxycinnamic acids (Figure 4).

### 2.2. Biological Activity

#### 2.2.1. H_2_O_2_ Induced Toxicity in V79 Cells

Cellular viability can be assessed by evaluating mitochondrial function. The mitochondrial dehydrogenases of viable cells, in contrast to dead cells, cleave the tetrazolium ring of the yellow MTT to yield purple formazan [16]. In order to evaluate the potential protective effects of the extracts, V79 cells were exposed to 37.5 µM H_2_O_2_ for 30 min, because under these conditions cellular viability was reduced by ca. 50% (data not shown).

#### 2.2.2. Effects of Kale and *P. brassicae* Extracts on V79 Cells Viability

The methanolic extracts of kale and *P. brassicae* materials were tested in concentrations equivalent to 0.22 to 135 mg of dried material/mL. According to the quantification of phenolic compounds (Table 1) this sample concentrations correspond to 0.83 to 510.25 µg/mL of phenolic compounds for kale extracts, 0.06 to 36.65 µg/mL for *P. brassicae* larvae and 0.01 to 6.66 µg/mL for their excrements. 

**Figure 5:**
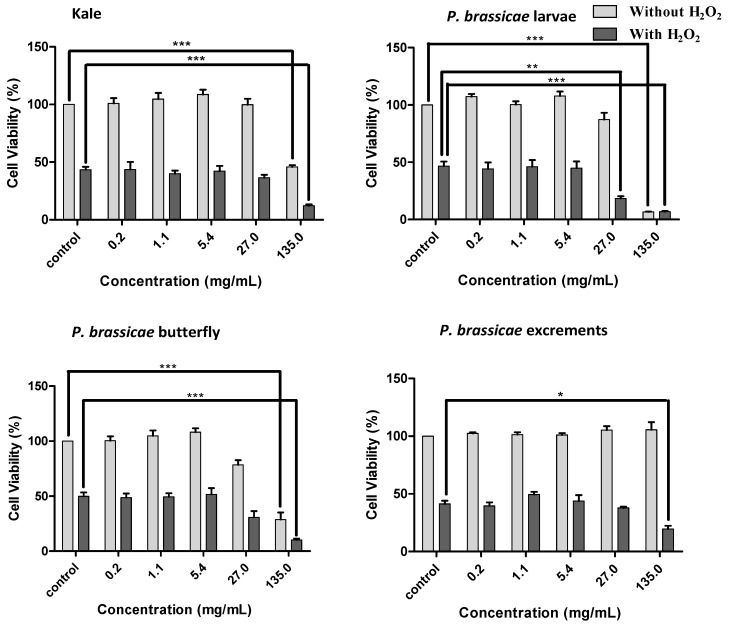
Effect of *B. oleracea* var. *acephala* and *P. brassicae* materials methanolic extracts on V79 cells viability with and without H_2_O_2_-induced oxidative stress. Values show mean ± SE (n = 4). Mean values were significantly different compared with the respective control (* *p <* 0.05, ** *p* < 0.01 and *** *p* < 0.001).

In general, extracts were cytotoxic to V79 cells at the highest concentration tested (135 mg/mL, *p* < 0.001) (Figure 5). For this concentration, cellular viability was ca. 46% for kale, 7% for larvae and 29% for butterflies, as ascertained by the results for MTT reduction. *P. brassicae* larvae and butterfly methanolic extracts already showed a tendency to be toxic to V79 cells at 27 mg/mL (Figure 5). *P. brassicae* excrements extract showed MTT reduction higher than 96% for all concentrations tested.

**Figure 6:**
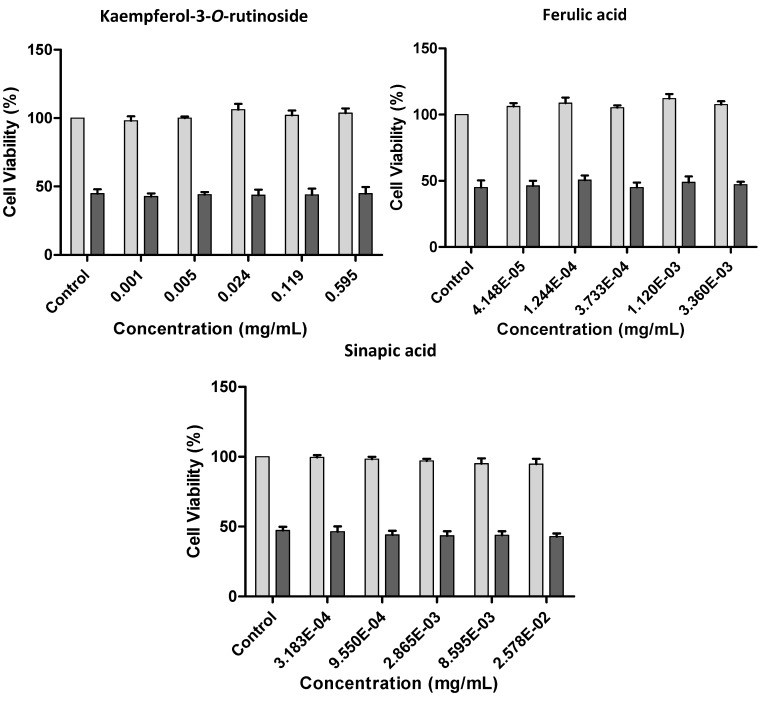
Effect of kaempferol-3-*O*-rutinoside, ferulic and sinapic acids on V79 cells viability with and without H_2_O_2_-induced oxidative stress. Values show mean ± SE (n = 4).

In order to assess the possible role of glycosylated flavonoids, kaempferol-3-*O*-rutinoside, a commercially available flavonoid glycoside closely related to the ones encountered in the extracts, was tested at 1–595 µg/mL (1.7–1,000 µM), which correspond to concentrations representative of kaempferol derivatives in the extracts (Figure 6). The same was done considering the hydroxycinnamic acids found only in *P. brassicae* larvae and excrements extracts: ferulic acid at 0.042–3.36 µg/mL (0.2–17.3 µM) and sinapic acid at 0.32–25.78 µg/mL (1.4–115.1 µM) were evaluated. None of the assayed compounds was toxic to V79 cells, as ascertained by the MTT assay (Figure 6). 

#### 2.2.3. Effect of Kale and *P. brassicae* Extracts on Cellular Hydrogen Peroxide-Induced Toxicity

To evaluate the protective effect of kale and *P. brassicae* materials, V79 cells were pre-treated with different extract concentrations before exposition to H_2_O_2_. None of the studied extracts provided protection. Furthermore, at the highest tested concentrations the extracts aggravated the toxicity induced by H_2_O_2_ (Figure 5, *p* < 0.001 for kale, larvae and butterfly extracts; *p* < 0.05 for excrements extracts). The deleterious effects of *P. brassicae* larvae extract were significant at 27.0 mg/mL (60%, *p* < 0.01). Kaempferol-3-*O*-rutinoside, ferulic and sinapic acids, assayed at the concentrations corresponding to their content in the matrices, didn’t prevent, nor aggravate the toxicity induced by H_2_O_2_ in V79 cells (Figure 6). 

#### 2.2.4. Effect of Kale and *P. brassicae* Extracts on Glutathione Homeostasis

**Figure 7:**
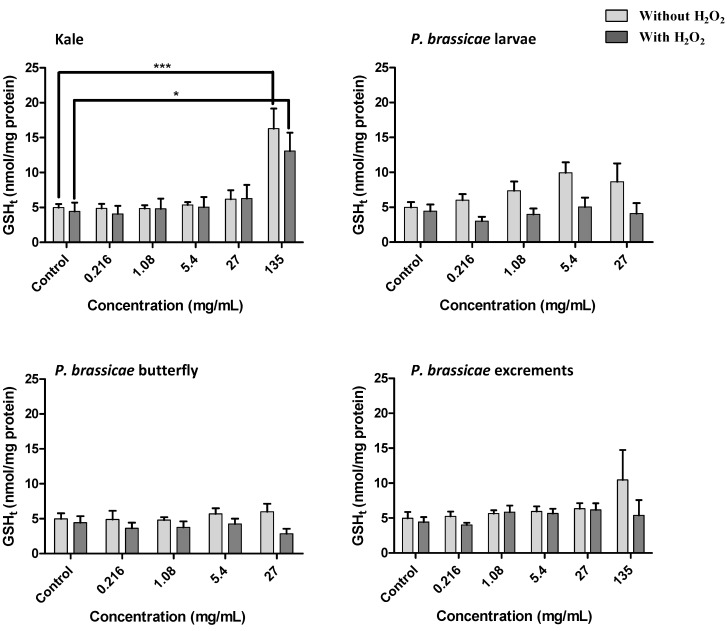
Effect of *B. oleracea* var. *acephala* and *P. brassicae* materials methanolic extracts on GSSG/GSH_t_ of V79 cells, after 24 h treatment, with and without H_2_O_2_-induced oxidative stress. Values show mean ± SE (n = 4). Mean values were significantly different compared with the respective control (* *p* < 0.05 and ** *p* < 0.01).

**Figure 8:**
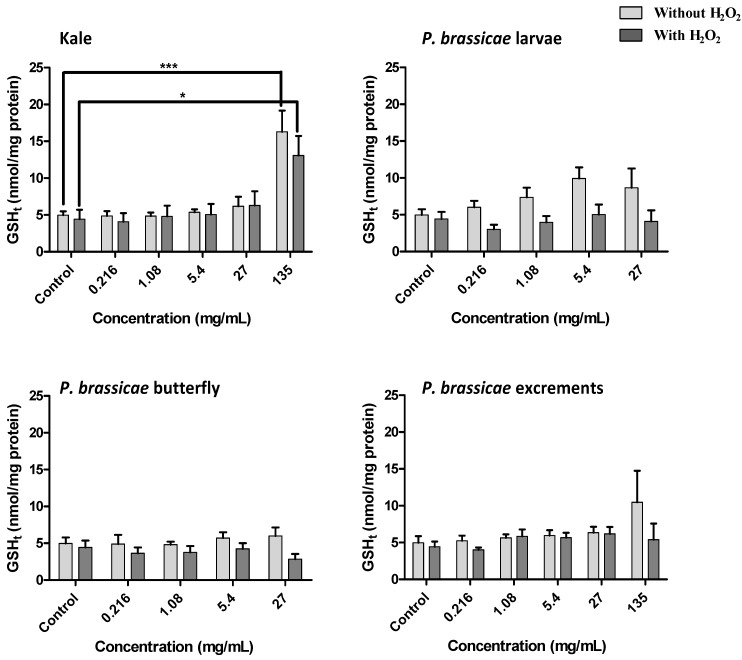
Effect of *B. oleracea* var. *acephala* and *P. brassicae* materials methanolic extracts on total glutathione content of V79 cells, after 24 h treatment, with and without H_2_O_2_-induced oxidative stress. Values show mean ± SE (n = 4). Mean values were significantly different compared with the respective control (* *p* < 0.05 and *** *p* < 0.001).

V79 cells exposed to the methanolic extracts, either in the presence or absence of H_2_O_2_, were evaluated for total glutathione (GSH_t_) and GSSG contents (Figure 7 and Figure 8). 

H_2_O_2_ treated cells showed a high GSSG/GSH_t_ ratio compared to quiescent cells (Figure 7). Kale and *P. brassicae* excrements extract lead to a significant disturbance on GSH homeostasis for the highest concentration tested (Figure 7). Comparing with the respective control, the increase in GSSG/GSH_t_ ratio induced by kale extracts was significant in quiescent cells and in cells exposed to H_2_O_2_ (Figure 7, *p* < 0.05), while for excrements extract this increase was only significant in quiescent cells (Figure 7, *p* < 0.01). Besides the increase in GSSG/GSH_t_ ratio induced by kale, the GSH_t_ levels were also significantly increased by this matrix (Figure 8, *p* < 0.001). This significant effect was also verified after exposition to H_2_O_2_ (Figure 8, *p* < 0.05). 

**Figure 9:**
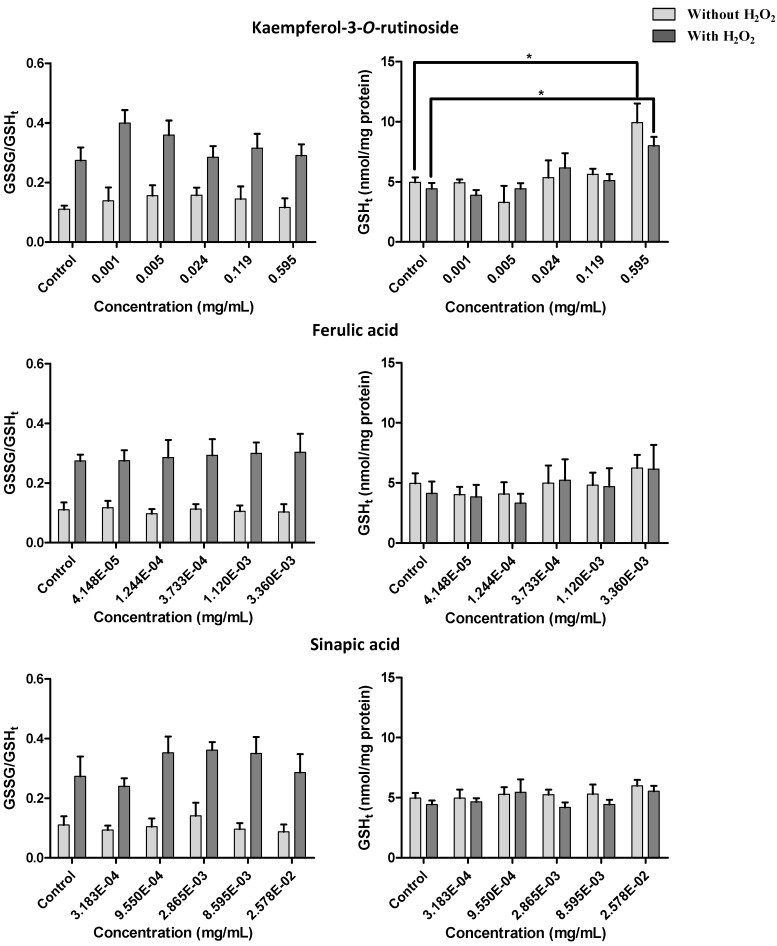
Effect of kaempferol-3-*O*-rutinoside, ferulic and sinapic acids on GSSG/GSH_t_ and on total glutathione content of V79 cells, after 24 h treatment, with and without H_2_O_2_-induced oxidative stress. Values show mean ± SE (n = 4). Mean values were significantly different compared with the respective control (* *p* < 0.05).

Considering both *P. brassicae* larvae and butterfly extracts, it was not possible to evaluate the glutathione levels at 135 mg/mL, once the cellular viability was already too low at this concentration (Figure 5). The GSSG/GSH_t_ ratio in V79 cells either with or without H_2_O_2_ treatment was not significantly affected by kaempferol-3-*O*-rutinoside, sinapic or ferulic acids tested at concentrations corresponding to their contents in the extracts (Figure 9). However, an increase of GSH_t_ levels was noticed with the highest tested concentration of kaempferol-3-*O*-rutinoside (*p* < 0.05), while ferulic and sinapic acids had no effect (Figure 9). 

## 3. Discussion

The richness of *Brassica* plants in phenolic compounds is well documented and it’s generally accepted that these secondary metabolites can contribute to the claimed beneficial health effects of vegetables belonging to this genus [2].

Another field of growing interest concerns the potential application of herbivores, that otherwise are considered plagues, as sources of bioactive compounds produced by their host plants. The ability of *P. brassicae* larvae to accumulate, metabolize and excrete phenolic compounds has been thoroughly studied by our group [6,8,9,10,11,12]. The phenolic composition of kale and *P. brassicae* materials methanolic extracts and their antioxidant potential against acute oxidative stress in a cellular model were now studied.

The HPLC-DAD analysis of the methanolic extracts of kale, *P. brassicae* larvae, excrements and butterflies demonstrates that, in general, the qualitative composition is similar to that of the aqueous extracts of the same materials previously described [6]. 

The free hydroxycinnamic acids (sinapic and ferulic acids) were not detected in the aqueous extracts previously characterized [6] but were reported before in *P. brassicae* larvae fed with *Brassicarapa* var. *rapa* [9,11] or with *B. oleracea* var. *costata* [10]. Their presence in the two referred *P. brassicae* larvae extracts was justified by their occurrence in high content in the host plants, which does not happen in kale. In the *P. brassica*e materials analyzed herein, the hydroxycinnamic acids can result from larvae metabolism, by deacylation of sugars in position 3 of flavonols or from the deglycosylation of hydroxycinnamic acyl gentiobiosides found in kale. Their presence in the methanolic extracts may be due to a more effective extraction by this solvent than by boiling water used in the previous work.

Concerning *P. brassicae* excrement methanolic extract, it contains non acylated glycosides of flavonoids that are not detected in kale leaves (Table 1) and may result from deacylation and other biotransformation reactions of the various acylated flavonol derivatives found in the host plant. In addition, flavonol sulfated derivatives were also noted in the excrements, as well as in the larvae methanolic extract. Conjugation with sulfate is a common phase II detoxification reaction. These compounds are found only in small amounts in *P. brassicae* larvae, indicating that they are mainly excreted by the insect.

Besides the qualitative differences, the amounts of phenolics varied greatly. The highest phenolics content was found in kale leaves, which contained fourteen times more phenolics than *P. brassicae* larvae and 77 times more phenolics than the excrements (Table 1). 

Although the relative amounts of non acylated flavonoid glycosides were similar among the three studied matrices (kale, larvae and its excrements), the other groups of phenolic compounds greatly differ. The hydroxycinnamic acyl gentiobiosides found in kale are absent in *P. brassicae* materials, being the opposite true for free hydroxycinnamic acids (Figure 4). Thus, hydrolysis of hydroxycinnamic acyl gentiobiosides by the larvae followed by the accumulation and excretion of the free acid seems to occur. In what concerns to acylated flavonoid glycosides, they seem to be mainly excreted by the larvae, although hydrolysis into the corresponding heteroside and the free hydroxycinnamic acid probably happens, contributing for the total amount of the latter in the larvae.

The antioxidant potential of kale and *P. brassicae* materials extracts has been previously demonstrated in cell-free systems [6]. However, the promising results obtained in those assays need to be confirmed in biological models more closely related to the *in vivo* situation. 

Lung fibroblasts are reported to respond to oxidative stress [17,18]. Hydrogen peroxide is a physiologic oxidant currently used in the evaluation of the antioxidant potential of phenolic compounds or plant extracts in cellular assays [19,20,21,22]. It can easily cross cell membranes, producing deleterious effects within the original or neighboring cells, being regarded as one of the principal intermediaries of cytotoxicity induced by oxidative stress [23].

Before being evaluated for their antioxidant potential the extracts were tested in quiescent cells in order to screen for toxic effects. With the exception of *P. brassicae* excrements, the extracts demonstrated to be toxic in concentrations corresponding to 135 mg of dried material/mL (Figure 5). Due to the chemical complexity of the extracts, it is not easy to point to which compounds are responsible for the displayed toxicity. However, in order to assess the possible role of phenolic compounds in the displayed toxicity kaempferol-3-*O*-rutinoside, and ferulic and sinapic acids were tested in the concentration range found in the extracts (Figure 6). It should be noticed that only the lowest concentrations of each tested compound is generally achieved in blood after consumption of polyphenolic rich foods (physiologic concentration about 0.1–1 µM) [24]. However, the consumption of polyphenolic enriched foods or supplements provide more phenolic compounds than that attained by a typical diet [25]. No toxic effects on V79 cells were observed with these compounds, even at the supra-physiologic concentrations. So, the cytotoxicity of the extracts is probably not due to their phenolic content. This result was partially expected considering that *P. brassicae* butterfly extract, with no phenolic compound determined, seems to be one of the most toxic extracts (Figure 5).

When evaluated for their antioxidant potential in V79 cells subjected to H_2_O_2_ induced acute oxidative stress, the extracts failed to protect and even aggravated the deleterious effects of this oxidative agent. With the exception of *P. brassicae* excrements, the observed potentiation of H_2_O_2_-induced toxicity can be partly attributed to the toxicity of the extracts (Figure 5). However, these results do not agree with the antioxidant potential exhibited before by aqueous extracts of the same matrices in several non-cellular assays [6]. In that work *P. brassicae* materials and host kale aqueous extracts exhibited scavenging capacity against superoxide and nitric oxide radicals. The lack of correlation between the two models can be explained taking into account that some activities of the compounds may not be evaluated in chemical systems, or the concentrations required to scavenge pro-oxidant species may be deleterious to the cells [26]. A similar behavior was observed with *B. oleracea* var. *costata*, closely related to kale [15]. In cellular models the phenolic ring of polyphenols can be metabolized by peroxidase to form pro-oxidant phenoxyl radicals which, in some cases, are sufficiently reactive to cooxidize GSH or NADH, accompanied by extensive oxygen uptake and reactive oxygen species formation [4].

Phenolics are known for their protective activity against the deleterious effects of H_2_O_2_ [27,28]. However, in this model, kaempferol-3-*O*-rutinoside, ferulic and sinapic acids, didn’t prevent nor aggravate the toxicity induced by H_2_O_2_ in V79 cells (Figure 6). The extracts were characterized by the presence of complex molecules, highly glycosylated, some of them being also acylated and sulfated. These molecules possess higher molecular weight and are more polar than aglycones. Thus, they may not manage to pass the cell membrane at amounts sufficient to act as antioxidants. In addition, the contribution of unidentified compounds to the observed cytotoxicity and potentiation of H_2_O_2_-induced toxicity cannot be ignored.

Glutathione is the most abundant non-protein thiol in living organisms, playing a crucial role in intracellular protection against toxic compounds, such as oxidative agents [29]. Specifically, GSH is involved in cellular defense against H_2_O_2_. The extracts by themselves seem to affect to some extent GSH homeostasis, leading to an increase of oxidized glutathione and, consequently, to an increase of the toxic effect of H_2_O_2_ (Figure 5 and Figure 7). On the contrary, kaempferol-3-*O*-rutinoside, sinapic or ferulic acids didn’t significantly affect the GSSG/GSH_t_ ratio in V79 cells either with or without H_2_O_2_ treatment (Figure 9).

Thus, none of the tested extracts, as well as standard compounds, provided protection against deleterious H_2_O_2_ effects in V79 cells. The cytotoxic effect of H_2_O_2_ has been found to be catalyzed by metal ions, especially iron and copper, and it has been reported that metal chelators are effective in preventing such damage [30]. Previous studies in V79 cells showed that polyphenols having *o*-hydroxyl groups are effective in protecting against H_2_O_2_ induced cytotoxicity, whereas compounds lacking one of the *o*-hydroxyl groups were ineffective [20]. The main phenolics in the analyzed extracts lack the catechol group, an important feature for metal chelation, which may, at least partially, explain our results.

Considering kale methanolic extract, it should be highlighted the significant higher GSH_t_ levels (over three times) found for the highest concentration tested (Figure 8). These results may suggest cells’ attempt to increase their levels of antioxidants, in order to overcome the aggression to which they were submitted. Among the three phenolic compounds tested, only kaempferol-3-*O*-rutinoside lead to an increase of GSH_t_ levels at the highest tested concentration (Figure 9). As so, flavonol derivatives seem to contribute for these results, once kale was clearly the richest matrix in terms of this kind of compounds (Figure 4 and Table 1). Flavonoids have already proved to induce γ-glutamylcysteine synthetase, the rate limiting enzyme involved in glutathione biosynthesis [29].

## 4. Experimental

### 4.1. Reagents

Ferulic and sinapic acids, quercetin-3-*O*-glucoside, kaempferol-3-*O*-rutinoside and isorhamnetin-3-*O*-glucoside were from Extrasynthése (Genay, France). Reagents for cell culture were obtained from Invitrogen (Gibco, Grand Island, NY, USA): Dulbecco’s modified Eagle’s medium (4.5 g/L glucose, with l-glutamine and pyruvate; DMEM), phosphate-buffered saline (PBS), trypsin (2.5%), penicillin (5,000 U/mL)-streptomycin (5,000 µg/mL) and fetal bovine serum (FBS). Folin-Ciocalteu reagent, reduced glutathione (GSH), oxidized glutathione (GSSG), glutathione reductase (GR) (EC 1.6.4.2) and other reagents were reagent grade and obtained from Sigma, (St. Louis, MO, USA) and Merck (Darmstadt, Germany). Water was treated in a Milli-Q (Millipore, Bedford, MA, USA) water purification system.

### 4.2. Samples

Wild *P. brassicae* larvae were collected in Bragança (Northeast Portugal) and taken to the laboratory to complete their life cycle, including oviposition in kale (*B. oleracea* var. *acephala*) leaves. Identification was performed by José A. Pereira, Ph.D. (CIMO). Larvae fed with kale ad libitum were allowed to develop until the fourth instar and kept without food for 12 h before freezing. The excrements were also collected and frozen. Other larvae were allowed to reach the adult stage, being collected less than 24 h after eclosion. *P. brassicae* (larvae, excrements and butterflies) and kale leaves were freeze-dried. The dried material was powdered and kept in a desiccator in the dark until analysis. Voucher specimens were deposited at Laboratory of Pharmacognosy from the Faculty of Pharmacy of Porto University.

### 4.3. Extract Preparation

Each sample (ca. 2.7 g) was thoroughly mixed with methanol (3 × 100 mL, at 600 rpm, for 1 h) and then filtered through a Büchner funnel. The extracts were concentrated to dryness under reduced pressure (40 °C), and redissolved in ca. 50 mL acidic water (pH 2 with HCl). For phenolics purification, the obtained solution was passed through a C18 non-end-capped (NEC) column (50 μm particle size, 60 Å porosity, 10 g of sorbent mass/70 mL of reservoir volume; Chromabond, Macherey-Nagel, Germany). The column was previously conditioned with 30 mL of methanol and 70 mL of acidic water. The phenolic fraction retained in the column was then eluted with methanol (ca. 50 mL). The extract was evaporated to dryness under reduced pressure (40 °C) and stored at −20 °C protected from light.

### 4.4. HPLC-DAD Phenolic Compounds Analysis

For HPLC-DAD analysis the dried extracts were dissolved in 2 mL of methanol and filtered through a 0.22 μm size pore membrane. Analyses were performed as previously described [6], using a Gilson HPLC-DAD unit and a Spherisorb ODS2 (25.0 × 0.46 cm; 5 μm particle size) column. Elution was developed with acetic acid 1% (A) and methanol (B), using the following gradient (1 mL/min): 0 min-10% B, 30 min-40% B, 35 min-60% B, 37 min-80% B, 50 min-94% B. Detection was achieved with a Gilson diode array detector. Spectroscopic data from all peaks were accumulated in the range 240–400 nm, and chromatograms were recorded at 330 nm. The different phenolic compounds were identified by comparing their chromatographic behavior and UV-vis spectra with authentic standards and with data previously obtained by our group, using the same analytical conditions [6]. In order to guarantee the identity of the peaks the samples previously characterized by LC-MS (references 6, 9 and 10), were newly injected and compared with the new ones.

Phenolic compounds quantification was achieved by the absorbance recorded in the chromatograms relative to external standards. Sinapic and ferulic acid derivatives were quantified as sinapic and ferulic acids, respectively. Since standards of several identified compounds were not commercially available, kaempferol, isorhamnetin and quercetin derivatives were quantified as kaempferol-3-*O*-rutinoside, isorhamentin-3-*O*-glucoside and quercetin-3-*O*-glucoside, respectively.

### 4.5. Cell Culture and Treatments

In order to study the antioxidant activity of the methanolic extracts of kale and *P. brassicae* materials, V79 hamster lung fibroblast line was used, following the method described before [31] with modifications. Cells were maintained and grown as a monolayer in culture plastic flasks (75 cm^2^). The culture medium was DMEM, containing 10% heat-inactivated fetal bovine serum, 100 U/mL penicillin, 100 µg/mL streptomycin and 1% non-essential amino acids. Cells were kept in an incubator at 37 °C with a humidified atmosphere of 95% air and 5% CO_2_.

The dried methanolic extracts of kale and *P. brassicae* materials were dissolved and diluted in medium containing 0.1% (v/v) dimethyl sulfoxide (DMSO). The final concentration of DMSO did not affect cellular viability.

To determine the effect of the extracts of kale and *P. brassicae* materials on V79 cells, viability and glutathione (total and oxidized) content were assessed 24 h after exposure. The potential protective effect against oxidative stress induced by H_2_O_2_ was also evaluated. For this purpose, in order to establish the H_2_O_2_ levels that resulted in 50% cell death, V79 cells were exposed to 37.5, 75.0 and 150 µM (final concentration) H_2_O_2_, for different periods (30 and 60 min). Attending to the results obtained (data not shown), 30 min exposure to 37.5 µM H_2_O_2_ was selected. So, cells were pre-treated for 24 h with the extracts and then exposed to H_2_O_2_ according to the selected conditions before determination of cellular viability and glutathione content.

### 4.6. Cell Viability

MTT was measured as described before by Sousa and collaborators [15]. Briefly, after cells exposure to extract, or extract plus H_2_O_2_, the medium was removed and the cells were incubated for 30 min, at 37 °C, with culture medium containing 0.5 mg/mL MTT. Afterwards, the solution was removed and formazan crystals were solubilized with 250 µL DMSO. The resulting purple solution was measured spectrophotometrically at 570 nm. Data are presented as the percentage of MTT reduction of treated cells relative to control, either with or without H_2_O_2_. Kaempferol-3-*O*-rutinoside and ferulic and sinapic acids were assayed in the same conditions of the extracts. Four independent assays were conducted, each one of them in quadruplicate.

### 4.7. GSH_t_ and GSSG Determination

The cellular glutathione (GSH_t_) levels were determined by the 5,5'-dithiobis(2-nitrobenzoic acid) (DTNB)-GSSG reductase recycling assay after protein precipitation with perchloric acid, as described before [15]. Oxidized glutathione (GSSG) was determined after sample pre-treatment with 2-vinylpyridine. Four independent assays were performed.

### 4.8. Measurement of Protein Content

Protein content was measured using Lowry method with bovine serum albumin as standard, as previously described [32].

### 4.9. Statistical Analysis

Comparisons were performed by two-way analysis of variance (ANOVA), with the Bonferroni *post hoc* test, using GraphPad Prism 5 software.

## 5. Conclusions

This study is the first report on the cell effect of *P. brassicae*. Also the activity of its host plant, kale, was evaluated for the first time in V79 cells. The results showed no protective activity under acute H_2_O_2_ cellular insult, implying that polyphenol rich extracts are not always beneficial under pro-oxidant conditions. Despite the ability of kale extract to increase glutathione levels, one of the most important cellular defenses against oxidative stress, the cytotoxicity of H_2_O_2_ was aggravated by the extract, in this cellular model. Indeed, the antioxidant properties of kale and *P. brassicae* materials previously observed in non-cellular assays and the results obtained in the present biological assay lack correlation. 

These results emphasize that the claimed antioxidant potential of extracts rich in phenolic compounds currently observed in cell free systems is not always confirmed in cellular models of induced oxidative stress. The results obtained also suggest that the industrial exploitation of kale or *P. brassicae* extracts as source of antioxidants needs care, as they may be especially harmful to health debilitated individuals.
